# *N*-(4-Methyl­benzo­yl)benzene­sulfonamide

**DOI:** 10.1107/S1600536810023974

**Published:** 2010-06-26

**Authors:** P. A. Suchetan, B. Thimme Gowda, Sabine Foro, Hartmut Fuess

**Affiliations:** a Department of Chemistry, Mangalore University, Mangalagangotri 574 199, Mangalore, India; b Institute of Materials Science, Darmstadt University of Technology, Petersenstrasse 23, D-64287 Darmstadt, Germany

## Abstract

In the title compound, C_14_H_13_NO_3_S, the conformation of the N—H bond in the C—SO_2_—NH—C(O) segment is *anti* to the C=O bond. The dihedral angle between the sulfonyl benzene ring and the S—N—C—O segment (r.m.s. deviation = 0.039 Å) is 77.1 (1)° and that between the sulfonyl and benzoyl benzene rings is 71.9 (1)°.

## Related literature

For background to our study of the effect of ring and side-chain substituents on the crystal structures of *N*-aromatic sulfonamides and for related structures, see: Gowda *et al.* (2009 ▶); Suchetan *et al.* (2009 ▶, 2010 ▶).
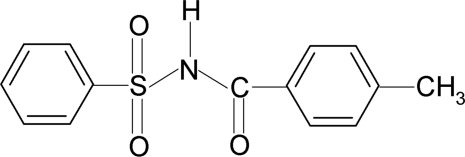


## Experimental

### 

#### Crystal data

C_14_H_13_NO_3_S
*M* *_r_* = 275.31
Triclinic, 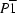

*a* = 5.5519 (6) Å
*b* = 10.541 (1) Å
*c* = 11.105 (1) Å
α = 85.654 (9)°
β = 83.667 (9)°
γ = 81.949 (9)°
*V* = 638.36 (11) Å^3^
*Z* = 2
Mo *K*α radiation
μ = 0.26 mm^−1^
*T* = 299 K
0.40 × 0.32 × 0.16 mm

#### Data collection

Oxford Diffraction Xcalibur diffractometer with a Sapphire CCD detector
Absorption correction: multi-scan (*CrysAlis RED*; Oxford Diffraction, 2009 ▶) *T*_min_ = 0.904, *T*_max_ = 0.960
4293 measured reflections
2595 independent reflections
2207 reflections with *I* > 2σ(*I*)
*R*_int_ = 0.017

#### Refinement

*R*[*F* ^2^ > 2σ(*F* ^2^)] = 0.038
*wR*(*F* ^2^) = 0.109
*S* = 1.08
2595 reflections
173 parameters
H-atom parameters constrained
Δρ_max_ = 0.24 e Å^−3^
Δρ_min_ = −0.30 e Å^−3^

### 

Data collection: *CrysAlis CCD* (Oxford Diffraction, 2009 ▶); cell refinement: *CrysAlis RED* (Oxford Diffraction, 2009 ▶); data reduction: *CrysAlis RED*; program(s) used to solve structure: *SHELXS97* (Sheldrick, 2008 ▶); program(s) used to refine structure: *SHELXL97* (Sheldrick, 2008 ▶); molecular graphics: *PLATON* (Spek, 2009 ▶); software used to prepare material for publication: *SHELXL97*.

## Supplementary Material

Crystal structure: contains datablocks I, global. DOI: 10.1107/S1600536810023974/ci5108sup1.cif

Structure factors: contains datablocks I. DOI: 10.1107/S1600536810023974/ci5108Isup2.hkl

Additional supplementary materials:  crystallographic information; 3D view; checkCIF report

## Figures and Tables

**Fig. 1.**
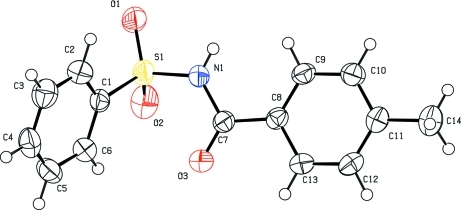
Molecular structure of the title compound, showing the atom-labelling scheme. Displacement ellipsoids are drawn at the 50% probability level.

## supplementary crystallographic information

### Comment

As a part of studying the effect of ring and the side chain substituents on the crystal structures of *N*-aromatic sulfonamides (Gowda *et al.*, 2009; Suchetan *et al.*, 2009, 2010), the crystal structure of *N*-(4-methylbenzoyl)benzenesulfonamide has been determined (Fig. 1). The conformation of the N—H bond in the C—SO_2_—NH—C(O) segment is *anti* to the C═O bond, similar to those observed in *N*-(benzoyl)benzenesulfonamide (II) (Gowda *et al.*, 2009), *N*-(benzoyl)-4-methylbenzenesulfonamide (III) (Suchetan *et al.*, 2010) and *N*-(4-chlorobenzoyl)-benzenesulfonamide (IV) (Suchetan *et al.*, 2009).

The molecules are twisted at the S—N bonds with the C—SO_2_—NH—C torsional angle of 67.4 (1)°, compared to the values of -66.9 (3)° in (II), 73.2 (2)° in (III) and 69.4 (2)° in (IV).

The dihedral angle between the sulfonyl-bound benzene ring and the S—N—C—O segment (r.m.s. deviation 0.039 Å) is 77.1 (1)°, compared to the values of 86.5 (1)° in (II), 76.5 (1)° in (III) and 75.7 (1)° in (IV).

The dihedral angle between the sulfonyl and the benzoyl benzene rings is 71.9 (1)°, compared to the values of 80.3 (1) in (II), 79.4 (1)° in (III), and 68.6 (1)° in (IV).

### Experimental

The title compound was prepared by refluxing a mixture of 4-methylbenzoic acid, benzenesulfonamide and phosphorous oxy chloride for 3 h on a water bath. The resultant mixture was cooled and poured into ice cold water. The solid obtained was filtered, washed thoroughly with water and then dissolved in a sodium bicarbonate solution. The compound was later reprecipitated by acidifying the filtered solution with dilute HCl. It was filtered, dried and recrystallized. Colourless needle-shaped single crystals of the title compound used in X-ray diffraction studies were obtained by slow evaporation of its toluene solution at room temperature.

### Refinement

H atoms were positioned with idealized geometry using a riding model with N–H = 0.86 Å, C–H = 0.93–0.96 Å and were refined with isotropic displacement parameters (set to 1.2 times of the *U*_eq_ of the parent atom).

### Crystal data

**Table:**

C_14_H_13_NO_3_S	*Z* = 2
*M**_r_* = 275.31	*F*(000) = 288
Triclinic, *P*1	*D*_x_ = 1.432 Mg m^−^^3^
Hall symbol: -P 1	Mo *K*α radiation, λ = 0.71073 Å
*a* = 5.5519 (6) Å	Cell parameters from 2679 reflections
*b* = 10.541 (1) Å	θ = 2.6–27.7°
*c* = 11.105 (1) Å	µ = 0.26 mm^−^^1^
α = 85.654 (9)°	*T* = 299 K
β = 83.667 (9)°	Needle, colourless
γ = 81.949 (9)°	0.40 × 0.32 × 0.16 mm
*V* = 638.36 (11) Å^3^	

### Data collection

**Table:**

Oxford Diffraction Xcalibur diffractometer with a Sapphire CCD detector	2595 independent reflections
Radiation source: fine-focus sealed tube	2207 reflections with *I* > 2σ(*I*)
graphite	*R*_int_ = 0.017
Rotation method data acquisition using ω and φ scans	θ_max_ = 26.4°, θ_min_ = 2.6°
Absorption correction: multi-scan (*CrysAlis RED*; Oxford Diffraction, 2009)	*h* = −6→6
*T*_min_ = 0.904, *T*_max_ = 0.960	*k* = −13→11
4293 measured reflections	*l* = −13→12

### Refinement

**Table:**

Refinement on *F*^2^	Primary atom site location: structure-invariant direct methods
Least-squares matrix: full	Secondary atom site location: difference Fourier map
*R*[*F*^2^ > 2σ(*F*^2^)] = 0.038	Hydrogen site location: inferred from neighbouring sites
*wR*(*F*^2^) = 0.109	H-atom parameters constrained
*S* = 1.08	*w* = 1/[σ^2^(*F*_o_^2^) + (0.0564*P*)^2^ + 0.1768*P*] where *P* = (*F*_o_^2^ + 2*F*_c_^2^)/3
2595 reflections	(Δ/σ)_max_ = 0.020
173 parameters	Δρ_max_ = 0.24 e Å^−^^3^
0 restraints	Δρ_min_ = −0.30 e Å^−^^3^

### Special details

**Table:**

Geometry. All e.s.d.'s (except the e.s.d. in the dihedral angle between two l.s. planes) are estimated using the full covariance matrix. The cell e.s.d.'s are taken into account individually in the estimation of e.s.d.'s in distances, angles and torsion angles; correlations between e.s.d.'s in cell parameters are only used when they are defined by crystal symmetry. An approximate (isotropic) treatment of cell e.s.d.'s is used for estimating e.s.d.'s involving l.s. planes.
Refinement. Refinement of *F*^2^ against ALL reflections. The weighted *R*-factor *wR* and goodness of fit *S* are based on *F*^2^, conventional *R*-factors *R* are based on *F*, with *F* set to zero for negative *F*^2^. The threshold expression of *F*^2^ > σ(*F*^2^) is used only for calculating *R*-factors(gt) *etc*. and is not relevant to the choice of reflections for refinement. *R*-factors based on *F*^2^ are statistically about twice as large as those based on *F*, and *R*- factors based on ALL data will be even larger.

### Fractional atomic coordinates and isotropic or equivalent isotropic displacement parameters (Å^2^)

**Table:**

	*x*	*y*	*z*	*U*_iso_*/*U*_eq_	
C1	0.0535 (3)	0.17087 (15)	0.37945 (14)	0.0370 (3)	
C2	0.2512 (3)	0.07861 (18)	0.39763 (17)	0.0491 (4)	
H2	0.3709	0.0944	0.4448	0.059*	
C3	0.2687 (4)	−0.03734 (19)	0.34486 (19)	0.0557 (5)	
H3	0.4008	−0.1001	0.3567	0.067*	
C4	0.0919 (4)	−0.06031 (17)	0.27507 (18)	0.0514 (5)	
H4	0.1050	−0.1383	0.2394	0.062*	
C5	−0.1047 (4)	0.03194 (19)	0.25778 (17)	0.0517 (5)	
H5	−0.2241	0.0157	0.2106	0.062*	
C6	−0.1262 (3)	0.14869 (17)	0.31001 (16)	0.0439 (4)	
H6	−0.2592	0.2110	0.2985	0.053*	
C7	0.1508 (3)	0.45512 (14)	0.24545 (14)	0.0362 (3)	
C8	0.2982 (3)	0.55415 (14)	0.18611 (13)	0.0345 (3)	
C9	0.4790 (3)	0.60283 (16)	0.23892 (15)	0.0393 (4)	
H9	0.5113	0.5748	0.3177	0.047*	
C10	0.6111 (3)	0.69266 (17)	0.17506 (15)	0.0429 (4)	
H10	0.7328	0.7235	0.2113	0.051*	
C11	0.5653 (3)	0.73765 (16)	0.05771 (15)	0.0403 (4)	
C12	0.3825 (3)	0.69012 (18)	0.00681 (16)	0.0471 (4)	
H12	0.3477	0.7197	−0.0712	0.056*	
C13	0.2508 (3)	0.60001 (17)	0.06897 (15)	0.0451 (4)	
H13	0.1291	0.5695	0.0324	0.054*	
C14	0.7134 (4)	0.83301 (19)	−0.01353 (19)	0.0553 (5)	
H14A	0.8409	0.7882	−0.0661	0.066*	
H14B	0.7844	0.8795	0.0415	0.066*	
H14C	0.6090	0.8920	−0.0613	0.066*	
N1	0.2026 (3)	0.40757 (13)	0.36175 (13)	0.0466 (4)	
H1N	0.3319	0.4262	0.3887	0.056*	
O1	0.1439 (3)	0.29602 (14)	0.55935 (12)	0.0705 (5)	
O2	−0.2229 (3)	0.37520 (13)	0.45410 (13)	0.0618 (4)	
O3	−0.0051 (2)	0.41356 (12)	0.19791 (11)	0.0493 (3)	
S1	0.02709 (9)	0.31703 (4)	0.45017 (4)	0.04709 (17)	

### Atomic displacement parameters (Å^2^)

**Table:**

	*U*^11^	*U*^22^	*U*^33^	*U*^12^	*U*^13^	*U*^23^
C1	0.0466 (9)	0.0344 (8)	0.0314 (8)	−0.0135 (7)	−0.0018 (6)	0.0008 (6)
C2	0.0481 (10)	0.0518 (10)	0.0501 (10)	−0.0113 (8)	−0.0125 (8)	−0.0001 (8)
C3	0.0556 (11)	0.0452 (10)	0.0633 (12)	−0.0003 (8)	−0.0039 (9)	0.0008 (9)
C4	0.0641 (12)	0.0386 (9)	0.0524 (11)	−0.0164 (8)	0.0059 (9)	−0.0079 (8)
C5	0.0555 (11)	0.0545 (11)	0.0510 (11)	−0.0219 (9)	−0.0081 (8)	−0.0105 (8)
C6	0.0453 (9)	0.0427 (9)	0.0453 (9)	−0.0096 (7)	−0.0076 (7)	−0.0014 (7)
C7	0.0448 (9)	0.0310 (8)	0.0330 (8)	−0.0035 (6)	−0.0065 (6)	−0.0027 (6)
C8	0.0391 (8)	0.0319 (8)	0.0313 (8)	−0.0004 (6)	−0.0035 (6)	−0.0018 (6)
C9	0.0429 (9)	0.0439 (9)	0.0313 (8)	−0.0054 (7)	−0.0071 (6)	0.0016 (6)
C10	0.0417 (9)	0.0476 (9)	0.0408 (9)	−0.0092 (7)	−0.0061 (7)	−0.0030 (7)
C11	0.0419 (9)	0.0359 (8)	0.0397 (8)	0.0000 (7)	0.0026 (7)	−0.0005 (6)
C12	0.0571 (11)	0.0495 (10)	0.0342 (8)	−0.0070 (8)	−0.0097 (7)	0.0074 (7)
C13	0.0524 (10)	0.0494 (10)	0.0366 (9)	−0.0127 (8)	−0.0145 (7)	0.0030 (7)
C14	0.0585 (12)	0.0500 (11)	0.0552 (11)	−0.0114 (9)	0.0036 (9)	0.0053 (9)
N1	0.0680 (10)	0.0420 (8)	0.0359 (7)	−0.0246 (7)	−0.0154 (7)	0.0053 (6)
O1	0.1269 (14)	0.0621 (9)	0.0333 (7)	−0.0439 (9)	−0.0218 (7)	0.0059 (6)
O2	0.0745 (10)	0.0493 (8)	0.0565 (8)	−0.0036 (7)	0.0148 (7)	−0.0111 (6)
O3	0.0544 (7)	0.0519 (7)	0.0459 (7)	−0.0172 (6)	−0.0166 (6)	0.0056 (5)
S1	0.0756 (3)	0.0393 (2)	0.0295 (2)	−0.0204 (2)	−0.00330 (19)	−0.00178 (16)

### Geometric parameters (Å, °)

**Table:**

C1—C6	1.381 (2)	C9—C10	1.384 (2)
C1—C2	1.382 (3)	C9—H9	0.93
C1—S1	1.7626 (16)	C10—C11	1.391 (2)
C2—C3	1.382 (3)	C10—H10	0.93
C2—H2	0.93	C11—C12	1.383 (2)
C3—C4	1.373 (3)	C11—C14	1.510 (2)
C3—H3	0.93	C12—C13	1.377 (2)
C4—C5	1.376 (3)	C12—H12	0.93
C4—H4	0.93	C13—H13	0.93
C5—C6	1.385 (3)	C14—H14A	0.96
C5—H5	0.93	C14—H14B	0.96
C6—H6	0.93	C14—H14C	0.96
C7—O3	1.209 (2)	N1—S1	1.6529 (15)
C7—N1	1.395 (2)	N1—H1N	0.86
C7—C8	1.487 (2)	O1—S1	1.4257 (14)
C8—C9	1.391 (2)	O2—S1	1.4340 (15)
C8—C13	1.393 (2)		
			
C6—C1—C2	121.09 (16)	C9—C10—C11	121.20 (16)
C6—C1—S1	119.75 (13)	C9—C10—H10	119.4
C2—C1—S1	119.13 (13)	C11—C10—H10	119.4
C3—C2—C1	119.19 (17)	C12—C11—C10	117.86 (15)
C3—C2—H2	120.4	C12—C11—C14	120.74 (16)
C1—C2—H2	120.4	C10—C11—C14	121.39 (16)
C4—C3—C2	120.29 (18)	C13—C12—C11	121.56 (16)
C4—C3—H3	119.9	C13—C12—H12	119.2
C2—C3—H3	119.9	C11—C12—H12	119.2
C3—C4—C5	120.12 (17)	C12—C13—C8	120.55 (16)
C3—C4—H4	119.9	C12—C13—H13	119.7
C5—C4—H4	119.9	C8—C13—H13	119.7
C4—C5—C6	120.56 (17)	C11—C14—H14A	109.5
C4—C5—H5	119.7	C11—C14—H14B	109.5
C6—C5—H5	119.7	H14A—C14—H14B	109.5
C1—C6—C5	118.75 (17)	C11—C14—H14C	109.5
C1—C6—H6	120.6	H14A—C14—H14C	109.5
C5—C6—H6	120.6	H14B—C14—H14C	109.5
O3—C7—N1	119.51 (15)	C7—N1—S1	123.07 (12)
O3—C7—C8	123.77 (14)	C7—N1—H1N	118.5
N1—C7—C8	116.70 (14)	S1—N1—H1N	118.5
C9—C8—C13	118.37 (15)	O1—S1—O2	119.65 (10)
C9—C8—C7	124.69 (14)	O1—S1—N1	103.47 (8)
C13—C8—C7	116.93 (14)	O2—S1—N1	109.71 (8)
C10—C9—C8	120.45 (15)	O1—S1—C1	109.18 (9)
C10—C9—H9	119.8	O2—S1—C1	108.24 (8)
C8—C9—H9	119.8	N1—S1—C1	105.73 (8)
			
C6—C1—C2—C3	0.2 (3)	C10—C11—C12—C13	−0.7 (3)
S1—C1—C2—C3	178.30 (14)	C14—C11—C12—C13	177.89 (17)
C1—C2—C3—C4	0.1 (3)	C11—C12—C13—C8	0.2 (3)
C2—C3—C4—C5	−0.3 (3)	C9—C8—C13—C12	0.8 (3)
C3—C4—C5—C6	0.2 (3)	C7—C8—C13—C12	−178.80 (16)
C2—C1—C6—C5	−0.4 (3)	O3—C7—N1—S1	−12.2 (2)
S1—C1—C6—C5	−178.42 (13)	C8—C7—N1—S1	168.97 (11)
C4—C5—C6—C1	0.2 (3)	C7—N1—S1—O1	−177.83 (14)
O3—C7—C8—C9	−179.72 (16)	C7—N1—S1—O2	−49.07 (16)
N1—C7—C8—C9	−0.9 (2)	C7—N1—S1—C1	67.44 (15)
O3—C7—C8—C13	−0.1 (2)	C6—C1—S1—O1	149.92 (14)
N1—C7—C8—C13	178.70 (15)	C2—C1—S1—O1	−28.17 (17)
C13—C8—C9—C10	−1.3 (2)	C6—C1—S1—O2	18.16 (16)
C7—C8—C9—C10	178.29 (15)	C2—C1—S1—O2	−159.93 (14)
C8—C9—C10—C11	0.8 (3)	C6—C1—S1—N1	−99.34 (14)
C9—C10—C11—C12	0.2 (3)	C2—C1—S1—N1	82.58 (15)
C9—C10—C11—C14	−178.37 (16)		
